# Automatic Identification of Individual Drugs in Death Certificates

**DOI:** 10.3233/SHTI190208

**Published:** 2019-08-21

**Authors:** Soon Jye Kho, Amit Sheth, Olivier Bodenreider

**Affiliations:** a Kno.e.sis Center, Department of Computer Science and Engineering, Wright State University, Dayton, Ohio, USA; b National Library of Medicine, Bethesda, Maryland, USA

**Keywords:** Death Certificates, Drug Overdose, RxNorm

## Abstract

**Background.:**

Establishing trends of drug overdoses requires the identification of individual drugs in death certificates, not supported by coding with the International Classification of Diseases. However, identifying drug mentions from the literal portion of death certificates remains challenging due to the variability of drug names.

**Objectives.:**

To automatically identify individual drugs in death certificates.

**Methods.:**

We use RxNorm to collect variants for drug names (generic names, synonyms, brand names) and we algorithmically generate common misspellings. We use this automatically compiled list to identify drug mentions from 703,106 death certificates and compare the performance of our automated approach to that of a manually curated list of drug names.

**Results.:**

Our automated approach shows a slight loss in recall (4.3%) compared to the manual approach (for individual drugs), due in part to acronyms.

**Conclusions.:**

Maintenance of a manually curated list of drugs is not sustainable and our approach offers a viable alternative.

## Introduction

Recent mortality trends show a substantial increase in drug overdose death rates in the United States. From 2010 to 2015, the rate of drug overdose death has increased from 12.3 to 16.3 per 100,000 in the U.S. population [1]. Researchers have devoted a significant amount of effort to describing drug overdose trends and to identifying the population at risk, as attempts to address this public health crisis [1–3].

Mortality data are a valuable source of information for establishing drug overdose trends. Causes of death are classified in accordance with the International Classification of Disease, Tenth Revision (ICD-10). ICD-10 codes X40-X49 identify unintentional drug poisoning or overdose deaths, while drug-specific overdose deaths are identified by the contributory causes of death indicated by “T” codes (e.g., T40.1 indicates death due to poisoning by, adverse effect of and underdosing of heroin). ICD-10 codes have been used to facilitate the drug overdose trends analysis [4–6].

While ICD-10 codes support consistent coding of the underlying causes of death, they do not provide enough granularity especially when it comes to reporting drug overdose at the level of individual drugs. While some drugs are assigned a unique ICD-10 code, most of them are not. For example, drug overdose cases caused by heroin and methadone are assigned distinct ICD-10 codes (T40.1 and T40.3 respectively), but drug overdose cases caused by fentanyl and tramadol are clustered together and assigned the same code (T40.4 - poisoning by synthetic narcotics). This issue also affects all the other opioids (T40.2), as well as barbiturates (T42.3) and benzodiazepines (T42.4).

To mitigate the granularity issue, researchers have utilized the literal portion of death certificates (i.e., a short textual description of the cause of death) to identify the contribution of a specific drug to drug overdose cases [7]. The death certificates in which a specific drug is mentioned can be retrieved and used for establishing specific drug overdose trends. Yet, identifying drug mentions in death certificates is not a trivial task as drug entities are often denoted by different terminology variants, including generic names, brand names and synonyms. Moreover, drug names are sometimes misspelled in death certificates. Therefore, it is important to identify these variants in order to have complete and accurate retrieval of death certificates in which drugs are mentioned.

**Figure 1 –:**
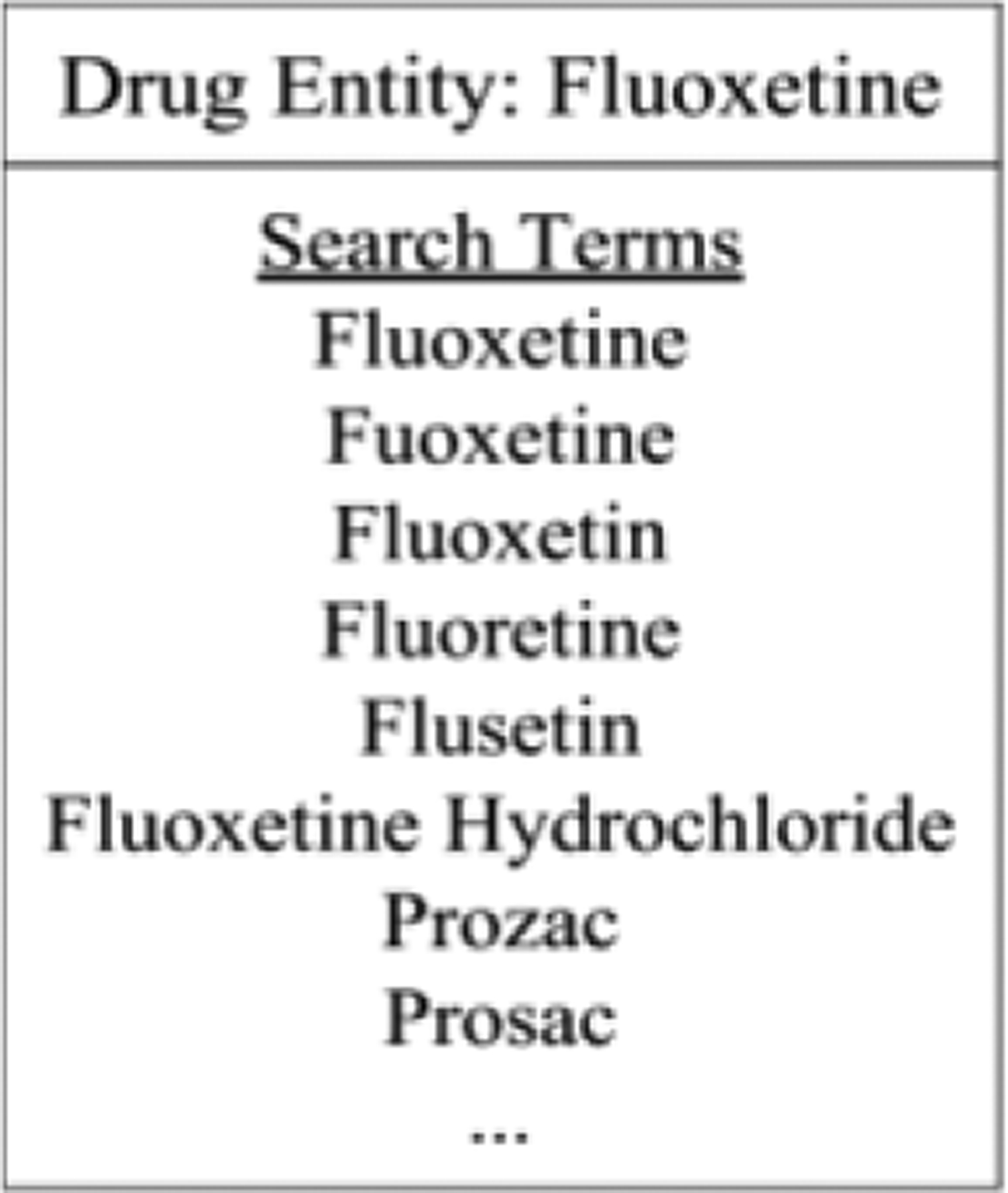
Example of drug entity in the Manually Curated List with the “principal variant” (top), and the set of terminology variants.

Trinidad et. al. [8] manually inspected death certificates over a 5-year period (2010–2014) and identified a list of search terms for drugs. These search terms were partially populated from Substance Abuse and Mental Health Services Administration’s (SAMHSA) Drug Abuse Warning Network (DAWN) Drug Reference Vocabulary (DRV) and complemented by drug mentions identified manually from the literal portion of death certificates. These search terms consist of various terminology variants, including synonyms, abbreviations, brand names, and misspellings. A study team trained in pharmacy and pharmaco-epidemiology then organized these search terms into their corresponding drug entities (around a “principal variant”), as illustrated in Figure 1. We refer to this list as the Manually Curated List (MCL).

This list covers a large number of drugs involved in the death of decedents but requires a significant amount of manual effort from domain experts for its curation. Manual curation is time-consuming and does not constitute a scalable and sustainable approach as new drugs are marketed and new variants appear in death certificates. Moreover, while terminology variants for a given drug entity were grouped together, the type of variant (e.g., brand name, misspelling) was not documented, making it impossible to study the specific contribution of each type of terminology variant.

The objective of this study is to explore an automated approach to generating a list of search terms for drugs to support the identification of individual drugs in death certificates. We assess the performance of our Automatically Compiled List (ACL) against the Manually Curated List (MCL) for the retrieval of death certificates. The main contribution of our work is to address the scalability and sustainability of drug identification in death certificates, by proposing an automated approach to generating the drug list.

## Methods

**Figure 2 –:**
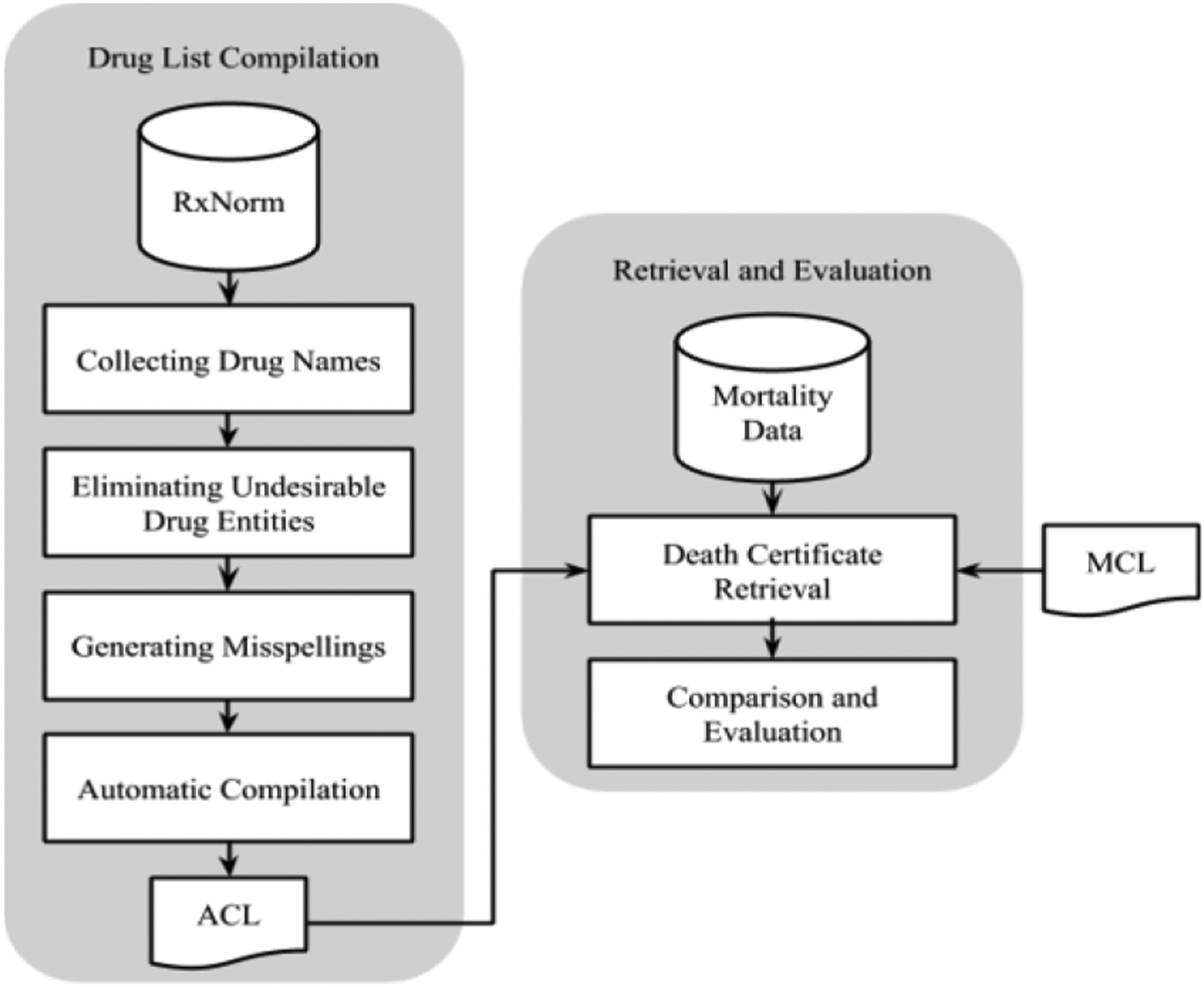
Overview of the methods.

We use RxNorm to collect variants for drug names (generic names, synonyms, brand names) and we algorithmically generate common misspellings. We use this automatically compiled list (ACL) to identify drug mentions from death certificates and compare the performance of our automated approach to that of the manually curated list (MCL) of drug names. (Figure 2).

### Drug List Compilation

The first stage focuses on compiling the list of search terms for drugs from RxNorm.

#### Collecting Drug Names from RxNorm

RxNorm is a normalized naming system for generic and branded drugs from a collection of commonly used public and private drug vocabularies [9]. The present study leverages RxNorm for populating drug names due to its comprehensive coverage of clinical drugs. We collected all names for drug entities from the following three RxNorm categories:

- Ingredients (IN) - a compound or moiety that gives the drug its distinctive clinical properties. Ingredients generally use the United States Adopted Name (USAN), e.g., *Fluoxetine*.
- Precise Ingredient (PIN) - a specified form of the ingredient that may or may not be clinically active. Most precise ingredients are salt or isomer forms, e.g., *Fluoxetine Hydrochloride*.
- Brand Name (BN) - a proprietary name for a family of products containing a specific active ingredient, e.g., *Prozac*.

The rationale for focusing on these three categories is that they are sufficient for identifying drug mentions in death certificates. While brand names generally correspond to prescription drugs (e.g., *oxycontin*), generic names for ingredients in RxNorm may also include substances for which no prescription drugs are available (e.g., *rofecoxib*). Other RxNorm categories, such as ‘Semantic Clinical Drug’ contain additional information (e.g., dose form and strength), usually not mentioned in death certificates and not essential for the retrieval of death certificates. We collect all synonyms from RxNorm for every drug name.

#### Eliminating Undesirable Drug Entities

RxNorm includes drug entities that are not of interest for identifying drugs in death certificates and will possibly generate false positives in retrieval, e.g., micro-organisms found in vaccines or used for allergy testing. We utilized semantic categorization (semantic type information) in the Unified Medical Language System (UMLS) [10] for filtering undesirable drug entities. All drug concepts from RxNorm are part of the UMLS Metathesaurus. For the purpose of retrieving death certificates, we only consider RxNorm concepts with the following UMLS semantic types:

- T109 - Organic Chemical
- T116 - Amino acid, peptide or protein
- T121 - Pharmacologic Substance
- T126 - Enzyme

In practice, we search in the UMLS Metathesaurus every RxNorm drug concept collected in the previous step and filter out those for which the semantic type is not one of the four listed above.

Another practical issue in identifying drug mentions in death certificates is that some drugs have brand names that correspond to frequently used English terms (e.g., *Prevail*, *Thrive*, *Today*). When found in death certificates, these terms generally do not denote a drug and will generate false positives in retrieval. In practice, we used the list of the top-5000 words from Word Frequency Data^1^ computed from the Corpus of Contemporary American English^2^ to filter out from our drug list any brand name that is present in this list.

#### Generating Misspellings

Some drug names are misspelled in death certificates. These misspellings indicate the mentions of drug entities but are often missed out in retrieval processes. We generated potential misspellings using an algorithm inspired by Pimpalkhute et. al. [11]. There are two phases in the algorithm: generation phase and filtering phase.

We first generated all variants with an edit distance of 1 (i.e., differing from the original by one character through insertion [*bupropion* / *buproprion*], deletion [*fluoxetine* / *fuoxetine*] or substitution [*Prozac* / *Pro*s*ac*]). We did not generate misspellings for chemical names (e.g., *1,1,2,2-tetrafluoroethane*) or drug names smaller than 5 characters (e.g. *Agar*, *Urea*, *Tums*) to avoid proliferation and ambiguity, respectively.

Misspelling generation has the potential to create a large number of variants. Further filtering (phoneme, lexical and semantic) is needed to select the most relevant misspellings. Phoneme filtering helps to reduce the spelling variants generated to a manageable number. Lexical and semantic filtering helps to avoid generating ambiguous variants that would likely generate false positives in retrieval.

1. ***Phoneme filtering*** helps select misspellings that sound like the original term, which are the most likely to be found in text. We use the metaphone algorithm to identify spelling variants of the original drug name with similar pronunciation (see [11] for details). The other spelling variants generated in previous steps are discarded.
2. ***Lexical filtering*** eliminates existing or potentially ambiguous variants. Short variants are discarded (variants of 4 characters or less) because short words tend to be ambiguous. Variants that correspond to existing English words are discarded because their mention in text most likely denotes an entity other than the drug. Finally, variants that correspond to existing drug names are discarded, because they are already covered by the main drug list.
3. ***Semantic filtering*** eliminates variants that correspond to existing biomedical concepts outside the drug domain for the same reason we eliminate variants that correspond to existing English words. In practice, we use the filter developed for eliminating undesirable drug entities (see above).

#### Automatically Compiled List of Drug Names

For the purpose of identifying drug mentions in death certificates, we organized the Automatically Compiled List (ACL) of drug names around RxNorm ingredients (IN). In practice, we grouped RxNorm precise ingredients (PIN) and brand names (BN), along with their synonyms and spelling variants, together with their corresponding ingredient. For example, mentions of *Prozac* (brand name for *fluoxetine*) and *fluoxetine hydrochloride* (precise ingredient, salt form of *fluoxetine*) are counted as mentions of *fluoxetine*. However, we keep track of the specific type of terminology variant (e.g., brand name) for each variant.

### Evaluation

We use this automatically compiled list to identify drug mentions from death certificates and compare the performance of our automated approach to that of the manually curated list of drug names. We use a corpus of death certificates from Washington State. This corpus spans a period of 14 years (from the year 2003 until 2016) and comprises a total of 703,106 death certificates. After indexing the death certificates with the *Elasticsearch* search engine, we used the automatically compiled list (ACL) and the manually curated list (MCL) to query the corpus of death certificates and retrieved one set for each list. Counts of death certificates are aggregated by ingredient (ACL query) or by principal variant (MCL query). For the purpose of comparing ACL and MCL, we normalized terms from the MCL to RxNorm using the RxNorm API.

## Results

### Drug List Compilation

#### Collecting and Filtering Drug Names from RxNorm

A total of 22,161 drug names are collected from RxNorm. After eliminating undesirable drug entities, 21,459 drug names remain (11,396 ingredients, 2714 precise ingredients, 5887 brand names and 1462 synonyms).

The 702 variants eliminated are described below.

- UMLS filtering: 689 RxNorm concepts were eliminated because their UMLS semantic type was outside the drug domain. Examples include *Adenine* (T114 Nucleic acid), *Air* (T167 Substance), *Apple Juice* (T168 Food), *Candida albicans* (T004 Fungus) and *Human poliovirus* (T005 Virus). Additionally, five obsolete drug names could not be mapped to the UMLS and were eliminated.
- Eight brand names corresponding to frequently used English terms (*Legend*, *Prevail*, *React*, *RID*, *Thrive*, *Today*, *Tomorrow* and *Triumph*).

#### Generating Misspellings

Our algorithm generated a total of 3,255,198 spelling variants, but most of them were eliminated during the filtering phase (Table 1). A total of 903,831 spellings variants was retained.

#### Automatically Compiled List of Drug Names

**Figure 3 –:**
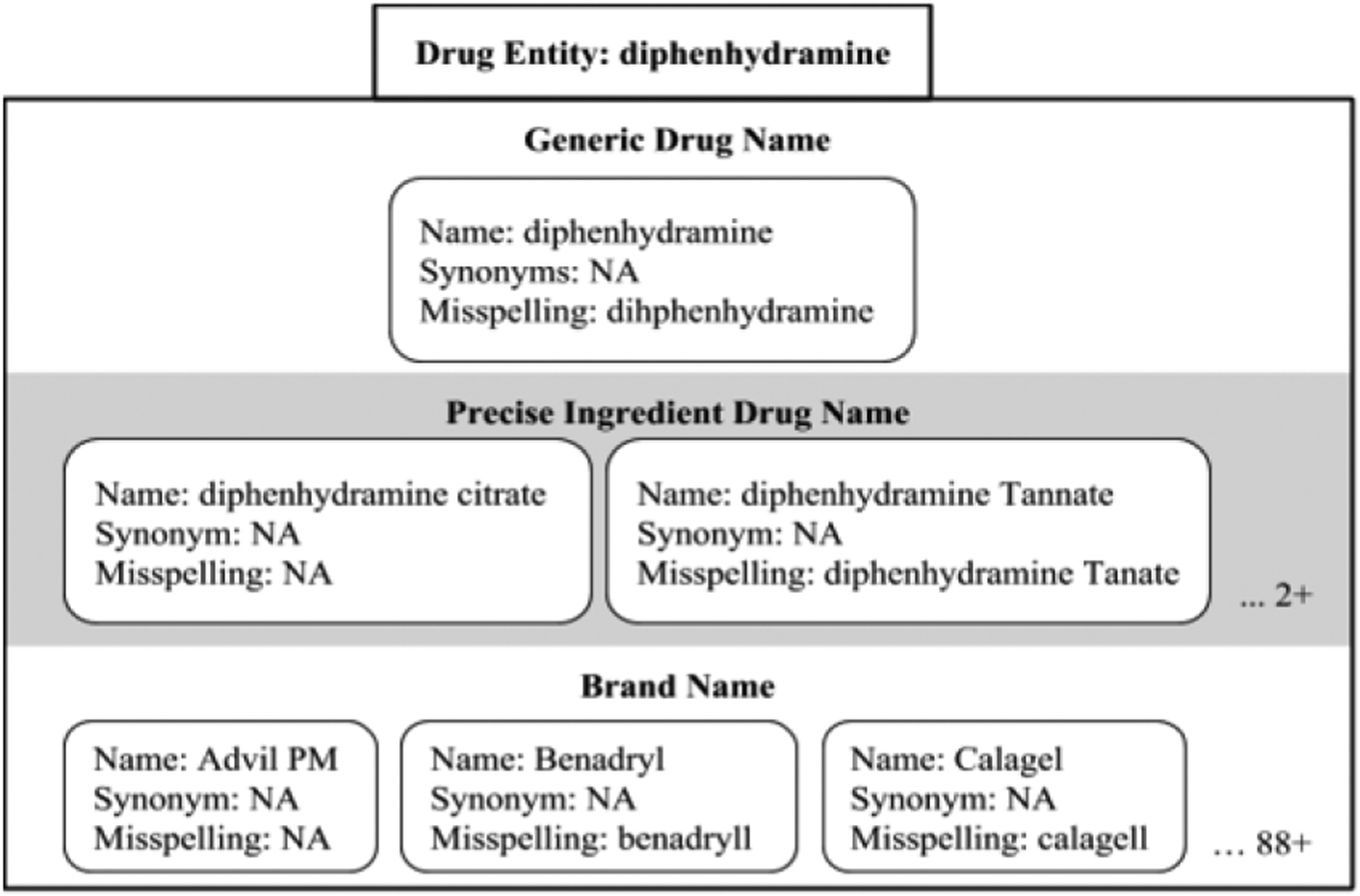
Example of a drug entity from ACL with different types of variants.

The Automatically Compiled List (ACL) of drug names, after eliminating undesirable drug entities and generating misspellings, comprises 925,290 variants organized around 11,396 main drug entities. Figure 3 shows the terminology variants for the drug entity *diphenhydramine*.

### Evaluation

#### Overall Retrieval of Death Certificates

A total of 37,215 unique death certificates were retrieved with the ACL query and 49,163 with the MCL query. Of these, 35,822 death certificates were retrieved by both queries, leaving 1,393 death certificates retrieved only with the ACL query and 13,341 retrieved only with the MCL query. The total number of death certificates retrieved with any query is 50,556, of which the ACL query retrieved 73.6% and the MCL query 97.2%. The difference in recall between ACL and MCL (23.6%) was expected, since MCL contains non-drug substances and drug classes, while ACL is restricted to drug names by construction.

#### Retrieval of Death Certificates for Individual Drugs

##### Quantitative evaluation.

There are 1654 drug entities present in both MCL and ACL. To assess variant generation in ACL, we specifically compared the number of death certificates retrieved for those drug entities in common between the two lists. A total of 35,674 unique death certificates were retrieved with the ACL query and 37,278 with the MCL query. Of these, 35,633 death certificates were retrieved by both queries, leaving 41 death certificates retrieved only with the ACL query and 1,645 retrieved only with the MCL query. The total number of death certificates retrieved with any query is 37,319, of which the ACL query retrieved 95.6% and the MCL query 99.9%. The difference in recall between both lists, when restricted to individual drugs, is only 4.3% (i.e., much lower than the loss in recall observed overall). Given the significant amount of manual effort involved in curating MCL, the slight loss in recall is indicative of a strong performance for the ACL query. Moreover, the fact that the ACL query retrieved 41 death certificates not retrieved with the MCL query (for variants including *alteplace*, *remicaide*, and *gentamycin*) demonstrates the benefit of a systematic, algorithmic approach to collecting drug names and spelling variants.

##### Qualitative evaluation.

To assess whether ACL and MCL would identify similar trends in drug-related mortality, we compared the top-20 drug entities retrieved by MCL and ACL in death certificates. Table 2 shows that both lists essentially identify the same top-20 drugs. Among the top-20 drug entities in both lists, 19 overlaps. (*Cholesterol* is inappropriately identified as a drug in ACL, but not MCL.) The ranking of these drug entities is also identical in both list, except for the permutation of *Citalopram* and *Alprazolam*, whose frequencies are very close.

## Discussion

### Specific Contribution of Variant Types

Table 3 shows the overall frequency of death certificates retrieved by each type of terminology variant from the ACL. While ingredient names account for the vast majority of drug mentions, brand names and misspellings also contribute to the retrieval of death certificates.

Table 4 presents the top-5 brand names mentioned in death certificates. Overall, the usage of brand names constitutes 3.6% (1323/37,215) of all drug mentions. One exception is *Coumadin* (926 mentions), the brand name of *Warfarin* (788 mentions).

### Error Analysis

The error analysis reveals the search terms that lead to loss in recall. Table 5 shows the top-10 drug entities which have the highest difference in retrieval performance. These drug entities explain 61.2% (1007/1645) of the loss in recall. Most of the search terms responsible for the loss in recall are abbreviations (e.g., *ETOH* for *ethanol*). This particular term is responsible for 52.9% (870/1645) of the loss in recall. Adding this one term into ACL would decrease the loss in recall from 4.30% to 1.97%.

Upon inspection of the top-20 drug entities, we observed that some drug entities (from both ACL and MCL) tend to generate false positive results, i.e., retrieve death certificates for which the drug mentioned is not the cause of death (e.g., *Iron*, mentioned in the context of ‘iron deficiency anemia’, not iron-related overdose). Most of these drug entities have the semantic type of ‘T196 Element’ (e.g. *iron*, *oxygen*, *gold*, *helium*) and could easily be eliminated if it is confirmed that they yield false positives.

### Sustainability

RxNorm is updated monthly. Since ACL is built programmatically, it can be easily updated when new versions of RxNorm are released. Unlike MCL, the updated ACL will identify mentions of new drugs in death certificates. The algorithm used for generating misspellings is fast and could be run easily on new versions of RxNorm.

### Limitations

Our study only focused on drug identification. Assessing whether drug mentions in death certificates actually correspond to drug-related deaths is beyond the scope of this investigation.

Substances mentioned in death certificates are sometimes non-drugs (e.g., *fumes*, *cigarette*, *asbestos*) or drug class names (e.g., *opiates*, *narcotics*, *antipsychotic*, *steroid*) and are not covered by RxNorm. If ICD-10 is not sufficient for coding these substances, other sources can be investigated (e.g., drug classifications systems).

In this preliminary retrieval study, we only included the 184 misspellings that appear in our corpus of death certificates. Restricting misspellings to this small subset would not support the identification of different spelling variants in another corpus of death certificates. However, given the performance of the search engine, using the complete list of misspellings would not be a major issue.

## Conclusions

In this study, we explored an automated approach to compiling a list of search terms for identifying drug mentions in death certificates. We showed that the automatically compiled list (ACL) has a competitive retrieval performance for individual drugs compared to the manually curated list (MCL), with only a slight loss in recall, and can reproduce similar drug overdose trend analysis results. Importantly, unlike the manual approach, our automated approach is dynamic, scalable and sustainable.

## Figures and Tables

**Table 1 -:** Number of variants eliminated at each step

Filtering step	Number of variants eliminated	Number of remaining variants	Examples of variants eliminated
Phoneme filtering	2,343,923	911,275	*azciximab, prozaw*
Short variant filtering	7132	904,143	*born, corhd, parox, dylan*
Existing English word filtering	242	903,901	*captain, watery, concert*
Existing drug name filtering	63	903,838	*butabarbital, nexavar, protamine*
Semantic types filtering	7	903,831	*phosphide, ostium, ocular*

**Table 2 -:** Top-20 drug entities identified by MCL and ACL.

MCL		ACL	
Drug entity	Freq.	Drug entity	Freq.
Ethanol	4561	Ethanol	3588
Methadone	3377	Methadone	3375
Methamphetamine	2964	Metamphetamine	2946
Heroin	2456	Heroin	2446
Cocaine	2171	Cocaine	2166
Oxycodone	2100	Oxycodone	2093
Warfarin	1712	Warfarin	1709
Morphine	1412	Morphine	1404
Hydrocodone	1043	Hydrocodone	1037
		*Cholesterol*	*970*
Diphenhydramine	955	Diphenhydramine	944
*Alprazolam*	*886*	*Citalopram*	*879*
*Citalopram*	*884*	*Alprazolam*	*876*
Diazepam	828	Diazepam	827
Oxygen	814	Oxygen	814
Nicotine	685	Nicotine	685
Amitriptyline	623	Amitriptyline	622
Acetaminophen	555	Acetaminophen	552
Iron	533	Iron	533
Hydromorphone	508	Hydromorphone	497
Fentanyl	469	Fentanyl	468

**Table 3 -:** Overall frequency of death certificates retrieved by each type of terminology variant.

Drug category	Variant type	Aggregated number of death certificates*		
Ingredient	Drug Name	35,916	36,007	Total = 37,215
	Synonyms	48		
	Misspellings	398		
Precise Ingredient	Drug Name	122	122	
	Synonyms	0		
	Misspellings	0		
Brand Name	Drug Name	1,299	1,323	
	Synonyms	1		
	Misspellings	25		

* Some death certificates are counted multiple times here when more than one drug is mentioned.

**Table 4 -:** Top-5 brand names mentioned in death certificates.

Brand name	Generic name	Number of death certificates retrieved
Coumadin	Warfarin	926
Plavix	Clopidogrel	57
Tylenol	Acetaminophen	35
Adriamycin	Doxorubicin	33
Lovenox	Enoxaparin	16

**Table 5 -:** Top-10 drug entities which have the highest difference in retrieval performance. The search terms are only present in MCL and responsible for the loss in recall. Only the most significant search terms are listed, along with their terminology variant types.

Drug entity	Number of death certificate retrieved		Difference	Search terms (Number of death certificates not retrieved with ACL query)
	MCL	ACL		
*Ethanol*	4561	3588	973	Abbreviation - *ETOH* (870)
*Cannabis Sativa Subsp. Indica Top*	107	0	107	Synonym - *Marijuana* (29)
*1,1-difluoroethane*	78	0	78	Abbreviation - *Difluoroethane* (51)
*Alteplase*	37	5	32	Abbreviation - *TPA* (32)
*Dronabinol*	31	3	28	Abbreviation - *THC* (5)
*Isopropyl Alcohol*	33	7	26	Synonym - *Isopropanol* (10)
*Bupropion*	271	248	23	Misspelling - *Buproprion* (1)
*Cyclobenzaprine*	345	326	19	Misspelling - *Cyclobenzapine* (1)
*Methamphetamine*	2964	2946	18	Abbreviation - *Meth* (7)
*Dextromethorphan*	213	196	17	Misspelling - *Nyquil* (1)

## Footnotes

1
https://www.wordfrequency.info/free.asp

2
https://corpus.byu.edu/coca/
